# Chemical elements in *Elaeis guineensis* materials and derived oil

**DOI:** 10.1038/s41598-023-50492-8

**Published:** 2024-01-22

**Authors:** Hadee Thompson-Morrison, Fransisca Ariantiningsih, Sugesti Muhammad Arief, Sally Gaw, Brett Robinson

**Affiliations:** 1 School of Physical and Chemical Sciences, University of Canterbury, Christchurch, New Zealand; 2 Orangutan Information Centre, Medan, Sumatra Indonesia; 3 Present Address: Manaaki Whenua Landcare Research, Lincoln, New Zealand

**Keywords:** Element cycles, Metals, Environmental chemistry, Environmental impact

## Abstract

The production of oil palm (*Elaeis guineensis*) in Southeast Asia is vital to the economies of Indonesia and Malaysia. Both fertilisers and pesticides used in palm production can contain elevated concentrations of Trace Elements (TEs) which may accumulate in soils and leaf tissues of plants. We hypothesised that leaves from oil palms may be deficient in essential elements, while containing elevated concentrations of non-essential TEs commonly found in agrichemicals. Samples of plant materials (leaves and fruitlets) were collected from active and former plantations in Sumatra, Indonesia, and analysed for essential and non-essential elements. Indonesian palm oil samples were sourced in New Zealand and their elemental concentrations determined. Leaf materials from both active and abandoned production sites were deficient in N, K, S and Mo, while leaf materials from abandoned sites were deficient in P. These deficiencies may have been a contributing factor to the abandonment of production at these sites. Concentrations of non-essential elements were below or comparable to average plant concentrations and no evidence of contamination was found in plant tissues. Palm oil contained low concentrations of TEs, which did not pose any toxicity risks. However, Na and Al were present in concentrations of 1198 and 159 mg kg^−1^ respectively, which were higher than have been previously reported. Tropical oil palm production could benefit from the determination of bioaccumulation factors for fertiliser contaminants in *E. guineensis*, to limit the transfer of contaminants to plants and products if increased fertiliser applications were used to correct nutrient deficiencies.

## Introduction

The African oil palm (*Elaeis guineensis* Jacq.) is an economically important crop, producing the world’s most consumed vegetable oil^1^. Palm oil represents 10% of Indonesia’s exports and 5% of Malaysia’s, with these two countries being the world’s major cultivators of *E. guineensis*^2^. A 2016 assessment determined that 45% of harvested *E. guineensis* plantation land in Southeast Asia had been converted from tropical forests since 1989^3^. Tropical forest soil is typically nutrient-poor and requires inputs of both capital and maintenance nutrients through fertilisers to support production^4,5^. The essential elements of most importance for *E. guineensis* are N, P, K, Mg and B^6^. Inadequate supply of these elements through fertilisers can lead to yield reductions in *E. guineensis* production, particularly on acidic and weathered tropical soils such as those from Indonesia and Malaysia^6,7^. Plant deficiencies can be assessed through the leaf concentrations of essential elements.

Fertiliser-borne trace elements (TEs) can accumulate in soil to concentrations that exceed guideline values^8^. Pesticides and other agrichemicals used to support production are also potential sources of TEs^6,9,10^. If soil-accumulated TEs are taken up by plants, this has potential for products to exceed food safety standards^8,11^. The transfer of TEs into plant products is a function of plant TE uptake, surface deposition and post-harvest contamination. The root absorption of TE ions in soil solution occurs via the symplastic and apoplastic pathways, followed by xylem transport to leaves and potential phloem transport to fruit tissues^12^. Spray residues containing TEs can sorb to plant leaves after direct pesticide applications, while deposition of soil containing TEs onto plant parts used for manufacturing can lead to plant products containing TEs^13^. Trace elements can also enter products due to contamination from machinery and equipment during manufacturing, processing and storage post-harvest^14,15^. The uptake of ions from soil solution is governed by its bioavailability: high total concentrations of TEs in soil do not always correspond with high uptake^16^. Bioavailability is a function of the solubility and speciation of the TE in soil, the presence of competing ions as well as the receptor organism^12^. Leaf concentrations—particularly pinnae concentrations in *E. guineensis*—of essential elements are better indicators of plant nutrition than the total soil concentration^17^. The presence of TEs in vegetable oils as a result of plant uptake and transport is typically limited due to their partitioning away from the lipid phase in plants^18^. This is supported by no identification of concentrations of TEs in processed palm oils at which breached food safety standards^19^. Although some TEs may be present in crude palm oil, these are removed during refining processes^20–22^.

Concurrent research on *E. guineensis* plantation soils from Indonesia^23^ determined that the soils analysed were deficient in essential nutrients N, P, K, Mg and Mo, and elevated above background levels in TEs Cu, Zn, As and Pb. Furthermore, the soils analysed were acidic and TEs were more mobile compared to other arable systems. In the current work, we analysed *E. guineensis* plant materials—leaves, rachides, endosperms and mesocarps—from the same plantations as well as the *E. guineensis* product palm oil for with a specific focus on plant-essential nutrient and TE composition.

We hypothesise that *E. guineensis* leaf concentrations of N, P, K, Mg and Mo will be deficient and below concentrations of 2.3%, 1400 mg kg^-1^, 7500 mg kg^-1^, 2000 mg kg^-1^ and 0.3 mg kg^-1^, respectively. Furthermore, we hypothesise that concentrations of Cu, Zn, As and Pb may be elevated in plant tissues. We hypothesise that palm oil will contain inconsequential levels of all elements below tolerable upper limits for food safety. This research aims to determine whether plants are deficient in essential nutrients and elevated in TEs, and thus the benefits and risks of using *E. guineensis* products with specific respect to chemical elements.

## Methods

### Site descriptions and sample collection

Plant material samples were collected from three *E. guineensis* plantations in Sumatra, Indonesia in May 2019. All plantations shared a common soil type of Acrisol (taxonomically related to Oxisols according to the USDA soil classification^24^). The sampling locality (approximately 3.5952° N, 98.6722° E) is reported in Thompson-Morrison et al.^23^. Materials collected included leaves, rachides and whole kernels, which were divided into mesocarp and endosperm prior to analysis. Plant material was cleaned with water and oven dried at 60 °C until a constant weight was obtained. All samples were ground prior to analysis.

The study was conducted in accordance with the relevant institutional, national, and international guidelines. Permissions were obtained from landowners for the collection of plant material and plants were identified by the landowners or farmers present during sampling. Samples were sent to the University of Canterbury in New Zealand under approved permits from both New Zealand’s Ministry for Primary Industries (permits #2019072466 for kernel material and #2018068845 for leaf material) and Indonesia’s Agency for Agricultural Quarantine (phytosanitary certificate #2019.2.0702.0.K10.E.000785 for kernel material and #2019.2.0702.0.K10.E.000728 for leaf material). Kernels were crushed and ground prior to export to ensure non-viability. Once in New Zealand, samples were stored and analysed in Physical Containment Level 2 approved facilities with kernels stored at ~ 4 °C.

Sites A and B were adjacent but separate sites that both bordered Gunung Leuser National Park and were abandoned after ca. 30 years of production. These sites were adjacent to each other and were separated northeast (Site A)/southwest (Site B). At the time of sampling, the sites were overgrown and had had no amendments applied for at least 3 years and had not been harvested throughout this time period. Previous to this, they were under corporate management and underwent regular harvesting. Sites A and B had slopes of approximately 12° in places and samples were taken based on practicality and accessibility from areas in which *E. guineensis* trees remained.

Site C was also near to Gunung Leuser National Park, however, was an active smallholder *E. guineensis* plantation. This site had been under production for ca. 20 years. Weeds were regularly cleared from the understory of this plantation using spray chemicals and fruit were regularly harvested. The slope of this site was < 4°.

Samples of refined palm oil were sourced from an Indonesian food manufacturer whose food products are marketed in New Zealand.

### Analytical methods

#### Pseudo-total element concentrations

We analysed pseudo-total element concentrations (hereafter referred to as elemental concentrations) of all samples using microwave digestion, following EPA Method 3051A^25^. A Ultrawave Single Reaction Chamber Microwave Digestion Unit was used. In each analysis, 0.20 g (± 0.01 g) was weighed into a digestion tube and 5 mL 70% HNO_3_ was added. Samples were pre-digested overnight and then digested in the Ultrawave at 230°C for 25 min^26^. Two reagent blanks and two standard reference materials^27,28^ were included with every 11 samples for quality assurance. Digestates were analysed by inductively coupled plasma-mass spectrometry along with standard reference materials^29^. Recoveries of SRMs ranged from 79–134% of certified values. A full list of SRM recoveries is available in Supplementary Materials Table S1. All concentrations reported are on a dry weight basis. The limit of quantification for analyses was 0.01 mg kg^-1^.

#### Total carbon and nitrogen

Total C and N concentrations of all samples were analysed by C and N analyser: CN828 Elemental Analysis by Combustion. Blanks and SRMs^30^ were included with samples for quality assurance.

### Bioaccumulation coefficients

Bioaccumulation coefficients (BACs) give an indication of a plant’s ability to accumulate heavy metals from soils^31^. They can be calculated as the plant / soil TE concentration quotient for each plant tissue using the equation^32^

1 $$BAC=\frac{TE \left(plant\right)}{TE \left(soil\right)}$$

Bioaccumulation coefficients were calculated for pinnae, endosperm and mesocarp tissue from each site. These were calculated for Cu, Zn, As, Sr, Cd and Pb using soil concentrations from these sites detailed in Thompson-Morrison et al.^23^. These TEs were chosen as they are common contaminants of agrichemicals used to support production^33^.

### Statistical Analysis

R (version 4.1.0) was used for all statistical analysis^34^. One-way parametric Analysis of Variance (ANOVA) was run to compare means of each sample type between site using the package multcomp^35^. Data was (natural) log-transformed where assumptions of normality were not met and non-parametric permutational ANOVA was used when assumptions of homoscedacity were not met. In most cases there were no differences between the results of the parametric and non-parametric ANOVA and the parametric test was considered robust. When significant differences were identified, Tukey’s Honest Significant Difference test was run to determine which sites these existed between.

## Results and discussion

### Elements in *E. guineensis* leaves (pinnae and rachides)

#### Essential elements

Deficiencies of N, K, S and Mo were present in pinnae samples from all sites, whereas P deficiency was present in pinnae samples from abandoned Sites, A and B (Table 1). The leaf tissue samples tended to contain higher concentrations of both essential and non-essential elements compared to fruit (kernel and mesocarp) tissue. This is consistent with plant physiology, given leaves are a water-sink and elements taken up via the roots are transported here via xylem flow^12^.

**Table 1:** Mean essential element concentrations in E. guineensis leaf material (pinnae and rachides) at each site (standard error of the mean in parentheses) compared with available literature values for deficiency, adequate growth and toxicity, where available.

Site			A (n = 10) *	B (n = 7)	C (pinnae n = 2, rachides n = 1)	Deficient ^+^	Adequate ^+^	Excessive/toxic^+^
Pinnae	Macro-elements	C (%)	48(0.35)^a^	47(0.20)^a^	48(0.15)^a^	–	–	–
		N (%)	1.4(0.090)^a^	1.3(0.077)^a^	1.9(0.010)^b^	< 2.3	2.4–2.8	> 3.0
		P	1102(49)^a^	1054(27)^a^	1689(144)^b^	< 1400	1500–1800	> 2500
		K	9266(982)^a^	7109(857)^a^	5350(249)^a^	< 7500	9000–12,000	> 16,000
		S	1729(119)^a^	1799(148)^a^	1804(24)^a^	< 2000	2500–3500	> 6000
		Mg	3139(376)^a^	2883(354)^a^	2476(316)^a^	< 2000	2500–4000	> 7000
	Micro-elements	B	21(3.1)^a^	24(1.8)^a^	12(5.5)^a^	< 8.0	15–25	> 40
		Mn	272(44)^a^	910(197)^b^	705(150)^ab^	< 35	50 ^†^	400–1000 ^‡^
		Fe	83(5.1)^a^	80(5.3)^a^	68(7.1)^a^	–	100 ^†^	–
		Cu	5.8(0.26)^a^	5.9(0.73)^a^	7.6(2.2)^a^	< 3.0	5.0–8.0	> 15
		Zn	25(1.4)^a^	24(1.6)^a^	23(0.68)^a^	< 10	12–18	> 80
		Mo	0.13(0.025)^ab^	0.060(0.010)^a^	0.24(0.040)^b^	0.10–0.30 ^‡^	0.50–0.80	10–50 ^‡^
Rachides	Macro-elements	C (%)	47(0.14)^a^	47(0.19)^a^	48^a^	–	–	–
		N (%)	0.20(0.024)^a^	0.21(0.037)^a^	0.61^b^	–	–	–
		P	462(52)^a^	687(184)^a^	1316^a^	–	–	–
		K	8969(1760)^a^	6204(876)^a^	8614^a^	< 13,000	13,100–16,000	> 16,000
		S	837(96)^a^	998(126)^a^	947^a^	–	–	–
		Mg	742(145)^a^	898(43)^a^	1117^a^	–	–	–
	Micro-elements	B	28(4.2)^a^	25(5.0)^a^	22^a^	–	–	–
		Mn	35(7.0)^a^	132(26)^b^	78^ab^	–	–	–
		Fe	63(17)^a^	61(12)^a^	19^a^	–	–	–
		Cu	2.4(0.17)^a^	2.3(0.22)^a^	2.3^a^	–	–	–
		Zn	10(1.1)^a^	13(2.1)^a^	7.8^a^	–	–	–
		Mo	0.20(0.048)^a^	0.16(0.031)^a^	0.020^a^	–	–	–

All values in mg kg^-1^ unless otherwise specified. Sites which share the same letter(s) for a single variable are not significantly different from each other.

*B n = 9.

^+^Values specific to *E. guineensis* growth for palms > 6 years old^17^.

^†^Typical values for adequate plant growth as none specific to *E. guineensis* were identified^12^.

^‡^Typical values for adequate plant growth as none specific to *E. guineensis* were identified^15^.

Mean concentrations of N, S and Mo in pinnae samples were in the deficient range for oil palm production at all sites. In contrast, P was deficient in 100% of samples at both of the abandoned sites (A and B) and mean K was deficient at Sites B and C (Table 1). Nitrogen deficiencies were present in all pinnae samples from the three sites. The mean N concentrations at Site C (1.9%) were comparable to those of palms grown on Malaysian Oxisols of 1.8%^14^. Across all sites, the N concentrations (1.3–1.9%) were lower than those of oil palms grown in Nigeria (2.1%) and Indonesia (2.3–2.7%)^7,14^. For the Indonesian palms, 2.3% N was considered deficient. Nitrogen deficiencies have also been reported in oil palms from Cameroon^36^ and primarily affect leaf production, size, colour and net assimilation rate^17^. With insufficient N, oil palms will suffer depressed growth which becomes increasingly exacerbated with age^17^.

Phosphorus in all pinnae samples from Sites A and B were in the deficiency range for *E. guineensis* (Table 1). These P concentrations were lower than those reported in Indonesia, Malaysian and Nigerian *E. guineensis* leaves of 1500–1700 mg kg^-1^^7^, 1210–1350 mg kg^-1^^14^ and 1280 mg kg^-1^^14^, respectively. The P concentration in leaves at Site C (1689 mg kg^-1^) was within the adequate range for *E. guineensis* and comparable to the value of 1700 mg kg^-1^ from *E. guineensis* also grown in Indonesia reported by Woittiez et al.^7^. The range of leaf P concentrations across the sites were comparable to those of New Zealand native species grown in low-fertility systems in nutrient-poor, non-fertilised soils^37^. This indicates the low nutrient status of the *E. guineensis* trees we sampled and is consistent with inadequate fertilisation. Sites A and B may have had low native P concentrations followed by insufficient capital and maintenance nutrients applied during production. Unlike N, P concentrations do not typically drop precipitously following site abandonment as P may bind to soil colloids and be retained in soils^38^. This indicates that P concentrations at Sites A and B were sub-optimal during production and may have been a contributing factor to low yields and subsequent site abandonment. Deficiencies of P in oil palm result in reduced growth rates with smaller fruit bunches, leaves and trunks, reducing yields from these trees^17^.

Rachis concentrations of K have been suggested as a more accurate indication of plant K status compared to pinnae concentrations as they provide a more accurate indication of fruit yield^39^. The K concentrations of the rachis samples range from 6204 mg kg^-1^ at Site B to 8969 mg kg^-1^ at Site A, indicating K deficiency (Table 1). Potassium deficiency is the most limiting nutrient factor affecting yield in oil palm production^17,40,41^. Like P, deficiencies of K in oil palm result in smaller fruit bunches as well as reduced number of fruit bunches per tree^17^. Insufficient K also leads to reduced disease resistance in oil palms^17^. Deficiency of this element has been attributed to inadequate K fertilisation of production soils which already have naturally low background levels^7,36^. Cost has been cited as a barrier to K fertilisation for Indonesian smallholders, as K fertilisers are not subsidised^7^. Concentrations of K in oil palm leaves from Indonesia and Malaysia have also been reported in deficient ranges of 6000–6300 mg kg^-1^^7^ and 5700–7600 mg kg^-1^^14^ respectively. Non-deficient levels of 8800 mg kg^-1^ were measured in Nigerian oil palm leaves^14^.

Among the three sites, 90% of pinnae samples from Site A, 71% of samples from Site B and 100% of samples from Site C were deficient in S. The S concentrations were comparable to those of Nigerian *E. guineensis* (1740 mg kg^-1^)^14^. Sulphur deficiency in oil palms has similar effects to N deficiency, i.e., stunted leaf growth and necrosis, as well as potential decreased resistance to *Cercospora* infection^17^. Boron was below the optimal range at Site C, and lower than reported concentrations for oil palm leaves also grown in North Sumatra (16.4–19.5 mg kg^-1^), which were expressing B deficiency symptoms^42^. Boron is the most significant TE for oil palm growth: with insufficient B, leaf development is severely affected and leaves become thin, short and brittle^17^. There is little information available on the role of Mo in oil palm growth^17^ and no studies reporting Mo concentration of *E. guineensis* leaves could be identified for comparison.

Magnesium, Fe, Cu and Zn in pinnae samples were within the adequate range for oil palm growth at all sites (Table 1). The Mg concentrations were comparable to those in leaves of *E. guineensis* grown in Indonesia and Nigeria of 2800–3000 mg kg^-1^ and 2330 mg kg^-1^^14^, respectively. Zinc concentrations were comparable to leaf concentrations from Malaysian *E. guineensis* plants of ca. 15–28 mg kg^-1^^43^. The Fe and Cu pinnae concentrations were within typical ranges contained in plant leaves^12^. Manganese was present in high concentrations in pinnae samples, however still within the range of concentrations likely to be present in plants^44^. Concentrations were significantly higher at Site B compared with Site A (Table 1). Toxicity thresholds for Mn are plant- and even cultivar-specific^12^, and no specific thresholds for oil palm have been identified. Considering differences between active and former production sites, the active site (C) was significantly higher in N and P than former sites (A and B) and significantly higher in Mo than Site B. This may be due to the Site C’s ongoing cultivation at the time of sampling, and thus more recent fertilisation.

Our leaf samples were collected from plantations with soils deficient in N, P, K and Mo^23^. Thus, it is unsurprising that plant concentrations of these essential elements are sub-optimal and likely to be limiting production, as plants source the majority of their nutrient requirements from bioavailable elements in soils through diffusion and mass flow^12^. These deficiencies are attributed to the conversion of acidic, low-fertility forest soils to production without the full amount of necessary inputs of both capital and maintenance nutrients^4,5^. As *E. guineensis* producers in Indonesia rely heavily on subsidised fertilisers, these deficiencies—particularly K for which no subsidised fertilisers are available—are commonplace, particularly in smallholder plantations^7,40^.

#### Non-essential trace elements

Most non-essential elements were present in concentrations less than the average concentrations for terrestrial plants (Table 2). The low concentrations of Cr (1.7–4.7 mg kg^-1^ in pinnae, 1.1–10 mg kg^-1^ in rachides) and Ti (1.5–1.8 mg kg^-1^ in pinnae, 0.60–0.87 mg kg^-1^ in rachides) indicate that the non-essential elements present in *E. guineensis* leaves were not a result of soil contamination of leaves. While Ti, Cr, Ni and Hg were present above average terrestrial plant concentrations^45^, they were below toxicity thresholds for leaf tissues^15^.

**Table 2:** Mean concentrations of non-essential trace elements in *E. guineensis* leaves (pinnae and rachides) at each site (standard error of the mean in parentheses) with available literature values for toxicity and tolerability in agronomic crops.

Site		A (n = 10)	B (n = 7)	C (pinnae n = 2, rachides n = 1)	Land plants average *	Excessive/toxic to plants^+^
Pinnae	Na	116(15)^a^	111(21)^a^	54(20)^a^	1200	–
	Al	46(5.4)^a^	44(4.5)^a^	30(9.2)^a^	500	–
	Si	1.3(0.019)^a^	1.3(0.014)^a^	1.3(0.035)^a^	200	–
	Ti	1.8(0.18)^a^	1.6(0.20)^a^	1.5(0.20)^a^	1	50–200
	Cr	4.2(0.92)^a^	4.7(1.1)^a^	1.7(0.52)^a^	0.23	5–30
	Co	0.071(0.010)^a^	0.093(0.020)^a^	0.04(< 0.01)^a^	0.5	15–50
	Ni	3.6(0.51)^b^	5.8(1.0)^b^	1.1(0.080)^a^	3	10–100
	As	0.030(< 0.01)^a^	0.031(< 0.01)^a^	0.025(< 0.01)^a^	0.2	5–20
	Sr	16(2.7)^a^	9.7(0.85)^a^	17(0.90)^a^	26	–
	Ag	< 0.01^a^	< 0.01^a^	< 0.01^a^	0.06	–
	Cd	0.027(< 0.01)^a^	0.023(< 0.01)^a^	0.020(< 0.01)^a^	0.6	5–30
	Te	< 0.01^a^	< 0.01^a^	0.29(0.29)^b^	–	–
	Cs	0.18(0.030)^a^	0.25(0.035)^ab^	0.41(0.045)^b^	0.2	–
	Ce	0.22(0.048)^a^	0.39(0.061)^a^	0.85(0.26)^b^	–	–
	Au	< 0.01^a^	< 0.01^a^	< 0.01^a^	0.002	–
	Hg	0.045(< 0.01)^a^	0.044(< 0.01)^a^	0.052(0.015)^a^	0.015	1–3
	Pb	0.64(0.060)^a^	0.76(0.12)^a^	0.62(0.15)^a^	2.7	30–300
Rachides	Na	173(19)^a^	170(14)^a^	192^a^	1200	–
	Al	18(2.7)^a^	24(3.6)^a^	13^a^	500	–
	Si	273(28)^a^	291(29)^a^	188^a^	200	–
	Ti	0.60(0.063)^a^	0.87(0.11)^a^	0.60^a^	1	–
	Cr	10(3.6)^a^	8.8(2.1)^a^	1.1^a^	0.23	–
	Co	0.134(0.040)^a^	0.14(0.030)^a^	0.020^a^	0.5	–
	Ni	5.3(1.6)^a^	5.5(1.2)^a^	0.50^a^	3	–
	As	< 0.01)^a^	< 0.01^a^	< 0.01^a^	0.2	–
	Sr	11(1.2)^a^	6.9(0.86)^b^	6.6^ab^	26	–
	Ag	< 0.01^a^	< 0.01^a^	< 0.01^a^	0.06	–
	Cd	0.014(< 0.01)^a^	0.010(< 0.01)^a^	0.010^a^	0.6	–
	Te	< 0.01^a^	< 0.01^a^	< 0.01^a^	–	–
	Cs	0.092(0.020)^a^	0.18(0.020)^b^	0.74^c^	0.2	–
	Ce	0.060(0.010)^a^	0.090(0.020)^a^	0.07^a^	–	–
	Au	< 0.01^a^	< 0.01^a^	< 0.01^a^	0.002	–
	Hg	< 0.01^a^	< 0.01^a^	< 0.01^a^	0.015	–
	Pb	0.27(0.051)^a^	0.35(0.080)^a^	0.080^a^	2.7	–

All values in mg kg^−1^. Sites which share the same letter(s) for a single variable are not significantly different from each other.

*^45^.

^**+**^^15^.

Mean concentrations of Na and Si were higher in rachides (170–192 mg kg^-1^ and 188–291 mg kg^-1^, respectively) compared with pinnae (54–116 mg kg^-1^ and 1.3 mg kg^-1^, respectively). Cadmium concentrations (0.020–0.027 mg kg^-1^ in pinnae, 0.010–0.014 mg kg^-1^ in rachides) were lower than those reported from Malaysian *E. guineensis* leaves (0.18–0.38 mg kg^-1^)^43^. Silver and Au were present in leaf tissues in concentrations < 0.1 mg kg^-1^ and pose no toxicity risks^46^.

The pinnae concentrations of Te at Site C (0.29 mg kg^-1^) were significantly higher than at Sites A and B, and higher than those reported in Angiosperm leaves of 0.017 mg kg^-1^^47^. Cerium was also significantly higher in pinnae from Site C compared to Sites A and B. There is a scarcity of data on Te and Ce in plants with which to compare our results. Pinnae from Site C contained significantly higher concentrations of Cs than Site A, while rachides from Site C were significantly higher in Cs than Sites A and B. Caesium concentrations in pinnae at Sites B and C (0.25 and 0.41 mg kg^-1^, respectively) and rachides at Site C (0.74 mg kg^-1^) were higher than average plant concentrations of 0.2 mg kg^-1^^45^. The higher Te, Cs and Ce at Site C may be due to current production, as these TEs may be present in agrichemicals which were being used at the site at the time of sampling.

### Elements in *E. guineensis* endosperms and mesocarps

#### Essential elements

The essential elements in the endosperm tissues (Table 3) were comparable to those in Malaysian and Nigerian *E. guineensis* endosperms, except for Mg and Fe which were both lower in our samples^43,48,49^. Endosperm tissues contained the highest P and Cu concentrations of all plant materials analysed. Mesocarp tissues tended to contain the lowest concentrations of most elements, particularly essential plant elements, of all plant materials analysed.

**Table 3:** Mean concentrations of essential elements in analysed *E. guineensis* endosperms and mesocarps from each site (standard error of the mean in parentheses).

	Site		A (n = 8)	B (n = 7)	C (n = 3)
Endosperm	Macro-elements	C (%)	61(0.37)^a^	62(0.41)^a^	60(1.0)^a^
		N (%)	1.4(0.10)^a^	1.3(0.12)^a^	1.3(0.19)^a^
		P	3618(131)^a^	3827(189)^a^	3125(16)^a^
		K	4870(109)^a^	5045(326)^a^	4932(414)^a^
		S	1665(79)^a^	1554(89)^a^	1456(114)^a^
		Mg	1958(76)^a^	1937(115)^a^	1574(105)^a^
	Micro-elements	B	67(7.8)^b^	37(3.8)^a^	52(6.4)^ab^
		Mn	136(18)^a^	285(69)^a^	157(20)^a^
		Fe	32(1.3)^a^	37(2.7)^a^	37(5.5)^a^
		Cu	17(0.73)^a^	16(0.8)^a^	17(1.7)^a^
		Zn	24(1.4)^a^	27(2.3)^a^	26(2.6)^a^
		Mo	0.24(0.033)^ab^	0.34(0.060)^b^	0.1(0.019)^a^
Mesocarp	Macro-elements	C (%)	66(1.7)^a^	70(1.0)^a^	64(4.4)^a^
		N (%)	0.29(0.02)^a^	0.31(0.044)^a^	0.42(0.035)^a^
		P	513(53)^a^	484(25)^a^	849(85)^b^
		K	4487(605)^ab^	3044(192)^a^	6876(1894)^b^
		S	981(92)^a^	815(29)^a^	994(74)^a^
		Mg	1166(180)^a^	803(84)^a^	1516(206)^a^
	Micro-elements	B	51(4.4)^a^	45(4.0)^a^	57(11)^a^
		Mn	5.6(0.70)^a^	10(2.4)^a^	14(5.5)^a^
		Fe	35(2.9)^b^	25(1.8)^a^	33(6.3)^ab^
		Cu	15(1.4)^a^	14(1.0)^a^	14(0.80)^a^
		Zn	4(0.47)^a^	3.7(0.443)^a^	8.2(1.1)^b^
		Mo	0.056(0.011)^a^	0.063(0.016)^a^	0.033(< 0.01)^a^

All values in mg kg^−1^ unless otherwise specified. Sites which share the same letter(s) for a single variable are not significantly different from each other.

Phosphorus and K in endosperm samples ranged from 3125–3827 mg kg^-1^ and 4870–5045 mg kg^-1^, respectively, within concentration ranges reported in Nigerian endosperms: 2600–4700 and 2770–6600 mg kg^-1^, respectively^49^. These concentrations were lower than those in Malaysian endosperms: 6520–6950 and 6930–7510 mg kg^-1^, respectively^48^. The concentrations of Mn in endosperm samples (136–285 mg kg^-1^) overlapped with the range reported in Malaysian endosperms (82–145 mg kg^-1^)^49^ but were lower than Mn concentrations reported in Nigerian samples of 410–610 mg kg^-1^^48^. Our Cu and Zn concentrations in endosperms tissue (16–17 and 24–27 mg kg^-1^, respectively) were similar to the ranges present in Malaysian endosperms of 16–18 and 25–36 mg kg^-1^, respectively^49^ and lower than those of Nigerian endosperm tissue^48^. Magnesium and Fe concentrations in our analysed samples were lower than those from Malaysia (2050–3060 mg kg^-1^ Mg and 43–52 mg kg^-1^ Fe)^48^ and Nigeria (2250–5540 mg kg^-1^ Mg and 110–220 mg kg^-1^ Fe)^49^. No data could be identified for comparison on concentrations of C, N, S, B or Mo in *E. guineensis* endosperm tissue. There were only two significant differences in concentrations of essential elements in endosperm tissues between sites: Site A was significantly higher in B than site B, while Site B was significantly higher in Mo than Site C.

The low concentrations in mesocarp tissues (Table 3) are typical of *E. guineensis*: as fruit ripens, essential elements in the mesocarp decrease due to dilution by growth or translocation into the endosperm^14^. Our samples were ripe when collected, and the concentration ranges of N, P, K and Mg—0.29–0.42%, 484–849 mg kg^-1^, 3044–6876 mg kg^-1^, 803–1516 mg kg^-1^, respectively—are comparable to those of ripe mesocarp: 0.33–0.41%, 440–530 mg kg^-1^, 2900–3700 mg kg^-1^, 1300–1500 mg kg^-1^, respectively^50^. Of the essential elements analysed, N, P, Mg, S Mn, Cu, Zn, and Mo were significantly higher in endosperms compared to mesocarps.

#### Non-essential trace elements

Excluding Na, Al, Si, Cr, Ni and Sr, all non-essential elements in *E. guineensis* endosperms and mesocarps were < 1.0 mg kg^-1^ (Table 4). Non-essential elements posed no toxicity risks for manufactured products. As palm oil is expressed or extracted from the mesocarp, the low concentrations in this plant material indicates that TE concentrations in palm oil are likely to be low and of no concern regarding food safety standards, unless it is contaminated during the extraction process. As with leaf concentrations, Cs was significantly higher in mesocarps from Site C. Other than this, there were no significant differences in mesocarps between sites. Low elemental concentrations are typical of endosperm tissue, as to reach this location of the plant elements must be carried by phloem transport and cross the placenta^51^. As with leaves, Ni was significantly lower in mesocarp tissue from Site C site compared with Sites A and B. Chromium was also significantly higher in mesocarps from Site A compared to Site B.

**Table 4:** Mean concentrations of non-essential elements in analysed *E. guineensis* endosperms and mesocarps from each site (standard error of the mean in parentheses).

Site		A (n = 8)	B (n = 7)	C (n = 3)
Endosperm	Na	223(33)^a^	128(15)^b^	112(24)^ab^
	Al	31(5.2)^a^	17(2.9)^a^	27(4.9)^a^
	Si	352(38)^a^	303(38)^a^	226(22)^a^
	Ti	0.33(0.016)^a^	0.39(0.051)^a^	0.30(< 0.01)^a^
	Cr	0.29(0.018)^b^	0.19(0.027)^a^	0.24(0.064)^ab^
	Co	0.013(< 0.01)^a^	0.014(< 0.01)^a^	0.010(< 0.01)^a^
	Ni	1.6(0.17)^b^	2.2(0.35)^b^	0.60(0.13)^a^
	As	< 0.01^a^	< 0.01^a^	< 0.01^a^
	Sr	5.0(0.59)^a^	4.6(0.32)^a^	3.9(0.44)^a^
	Ag	< 0.01^a^	< 0.01^a^	< 0.01^a^
	Cd	0.018(< 0.01)^a^	0.027(< 0.01)^a^	0.017(< 0.01)^a^
	Te	< 0.01^a^	< 0.01^a^	< 0.01^a^
	Cs	0.068(0.015)^a^	0.094(0.023)^a^	0.13(0.022)^a^
	Ce	0.16(0.028)^a^	0.080(0.014)^a^	0.14(0.023)^a^
	Au	< 0.01^a^	< 0.01^a^	< 0.01^a^
	Hg	< 0.01^a^	< 0.01^a^	< 0.01^a^
	Pb	0.031(< 0.01)^a^	0.021(< 0.01)^a^	0.027(< 0.01)^a^
Mesocarp	Na	213(29)^a^	164(9.4)^a^	180(27)^a^
	Al	35(6.3)^a^	28(3.1)^a^	31(8.6)^a^
	Si	328(22)^a^	320(12)^a^	313(38)^a^
	Ti	0.50(0.17)^a^	0.40(0.044)^a^	0.30(0.12)^a^
	Cr	3.8(0.28)^a^	2.6(0.39)^a^	4.0(1.0)^a^
	Co	0.070(< 0.01)^a^	0.066(0.010)^a^	0.060(0.012)^a^
	Ni	2.1(0.15)^a^	1.6(0.18)^a^	2.0(0.36)^a^
	As	< 0.01^a^	< 0.01^a^	< 0.01^a^
	Sr	4.5(0.64)^a^	4.4(0.47)^a^	6.3(1.3)^a^
	Ag	< 0.01^a^	< 0.01^a^	0.010(< 0.01)^a^
	Cd	0.014(< 0.01)^a^	0.011(< 0.01)^a^	0.010(< 0.01)^a^
	Te	< 0.01^a^	< 0.01^a^	< 0.01^a^
	Cs	0.24(0.074)^a^	0.28(0.071)^a^	0.73(0.082)^b^
	Ce	0.13(0.014)^a^	0.11(0.014)^a^	0.13(0.040)^a^
	Au	< 0.01^a^	< 0.01^a^	< 0.01^a^
	Hg	< 0.01^a^	< 0.01^a^	< 0.01^a^
	Pb	0.053(0.014)^a^	0.047(0.011)^a^	0.060(0.015)^a^

All values in mg kg^−1^. Sites which share the same letter(s) for a single variable are not significantly different from each other.

Mean Na in the endosperm samples ranged from 112–223 mg kg^-1^, within the range of 80–240 mg kg^-1^ from Malaysian kernels^49^ and below the range of 610–1200 mg kg^-1^ from Nigerian kernels^48^. Cadmium in our endosperm samples (0.02–0.03 mg kg^-1^) was lower than in samples from Malaysia (0.09–0.31 mg kg^-1^)^43^, while Pb (0.02–0.03 mg kg^-1^) was comparable to the below-detection concentrations of ≤ 0.05 mg kg^-1^ reported in Nigerian kernels^48^.

### Bioaccumulation coefficients

Most BACs for TEs in *E. guineensis* at the sites sampled were < 0.5 (Table 5). A BAC of ≥ 1 indicates that a plant may take up higher than typical concentrations of a TE from soil^31^. These results are consistent with low BACs of most non-essential TEs measured in other studies^52,53^ except for Sr, for which no BACs could be identified from literature.

**Table 5:** Bioaccumulation coefficients of TEs in plant tissues at each if the *E. guineensis* plantation sites sampled.

	Site A			Site B			Site C		
	Pinnae	Endosperm	Mesocarp	Pinnae	Endosperm	Mesocarp	Pinnae	Endosperm	Mesocarp
Cu	0.31	0.89	0.79	0.16	0.42	0.37	1.4	3.0	2.5
Zn	0.27	0.26	0.044	0.26	0.30	0.041	0.31	0.35	0.11
As	0.0079	< 0.0026	< 0.0026	0.0072	< 0.0026	< 0.0023	0.0027	< 0.0011	< 0.0011
Sr	0.67	0.21	0.19	0.69	0.33	0.31	0.35	0.81	0.13
Cd	0.19	0.13	0.10	0.26	0.31	0.13	0.24	0.20	0.12
Pb	0.040	0.0019	0.0033	0.045	0.0012	0.0028	0.022	0.0010	0.0021

Coefficients for As in endosperm and mesocarp tissues are presented as < their calculated value as As concentrations in these fruit tissues was < 0.01.

The BAC of Cu in all plant tissues at Site C was > 1, indicating *E. guineensi*s here contained high concentrations of Cu relative to the soils at this site. This may have been due to direct application of Cu-containing spray residues onto plant tissues, as this site was still under active management, however, this is unlikely as endosperm and mesocarp tissue had higher BACs relative to pinnae. Leaf tissues typically receive foliar spray applications while internal fruit tissues do not, therefore if this were the reason for the high BACs at this site, it is likely that endosperm and mesocarp BACs would be lower than that of pinnae tissues. While it is possible that Cu may be translocated from the leaf to the fruit following direct contamination of leaves with Cu spray, the higher BACs of fruit tissue relative to leaf tissue indicate that Cu was likely entering plants via root uptake from soil solution and was highly phloem-mobile. This is supported by the BACs for Cu in fruit tissues at Site A (0.89 and 0.79 for endosperm and mesocarp, respectively), which were higher than the pinnae BAC at this site. The high BAC of Cu in *E. guineensis* fruit tissues may result in high Cu concentrations in plant products, including oil and PKE, if production soils contain excessive Cu concentrations. This may present risks to human and animal health if food and fodder safety standards, respectively, for Cu are exceeded.

The BAC of Sr in pinnae at both Sites A and B (0.67 and 0.69, respectively) indicated that Sr was likely entering plant tissues *E. guineensis* at these sites via soils. The BAC of Sr at Site C (0.81) also indicated that Sr maybe phloem mobile and easily transported to fruit tissues. There is little published information on Sr BACs or transport in plants with which to compare these results.

As *E. guineensis* is cultivated in plantations with differing management styles, including smallholder, corporate- and government-owned^54^, the determination of BACs for fertiliser contaminants in *E. guineensis* grown under a range of conditions with differing agrichemical input rates may be of benefit to this production systems. This research may develop strategies to limit the transfer of contaminants to plants and products if increased fertiliser applications were used to correct nutrient deficiencies.

### Elements in palm oil

Unlike TEs, plant oils are not transported by xylem or phloem from leaves to fruit tissue but are synthesised from sugars in situ^18^. Most TEs occur as hydrophilic metal complexes in plants, partitioning away from oils in plant tissues^55^. As such, high TE concentrations in vegetable oils are not common. Elemental concentrations in refined palm oil were lower than food safety standards and posed no risk of elemental toxicity. However, this study did not analyse pesticide residues which may be present in oils. No elements in the oil we analysed exceeded tolerable upper limits or maximum dietary reference intakes (Table 6). This is consistent with other studies addressing the elemental composition of plant-derived oils, in which TEs were found either below detection limits or in concentrations ≤ 1.0 mg kg^-1^^56,57^.

**Table 6:** Mean concentrations of elements in refined palm oil (standard error of the mean in parentheses) with tolerable upper limits, where available, and concentration ranges reported in previous studies.

Element	Mean (mg kg^-1^) (n = 10)	Maximum consumed quantity (mg day^-1^) *	Tolerable upper limit (mg day^-1^) ^+^	Total ranges reported in other studies (mg kg^-1^)
Na	1198(155)	44	2300	30–533^ab^
Mg	3.1(0.41)	0.12	350	0.02–192^cabd^
Al	159(8.5)	0.59 (mg kg^-1^ bw wk^-1^) ^†^	1 (mg kg^-1^ bw wk^-1^) ^†^	1.9–31^ae^
P	3.5(0.077)	0.13	4000	8–47 ^f^
S	60(3.9)	2.2	–	–
K	155(11)	5.8	4500–4700 ^‡^	0.39–165^bd^
Ca	51(2.8)	1.9	2500	0.34–867^cabd^
Cr	0.080(0.019)	< 0.01	0.02–0.04 ^‡^	0.02–2.3^beg^
Mn	0.25(0.049)	< 0.01	11	0.24–12^abh^
Fe	2.6(1.3)	0.096	45	0.12–232^cfbdeh^
Co	0.019(< 0.01)	< 0.01	–	0.00–0.06^h^
Ni	0.031(0.010)	< 0.01	1	0.00–0.81^bgh^
Cu	0.048(0.018)	< 0.01	10	0.00–2.1^cfabeh^
Zn	< 0.01	< 0.01	40	0.05–15^bdh^
As	0.014(< 0.01)	< 0.01	–	≤ 0.03^gij^
Sr	0.22(< 0.01)	< 0.01	–	–
Zr	1.4(0.096)	0.052	–	–
Mo	0.021(0)	< 0.01	2	–
Ag	< 0.01	< 0.01	–	–
Cd	0.015(< 0.01)	< 0.0001 (mg kg^-1^ bw wk^-1^) ^†^	0.007 (mg kg^-1^ bw wk^-1^) ^†^	0.02–0.09^beg^
Te	0.010(< 0.01)	< 0.01	–	–
Au	< 0.01	< 0.01	–	–
Hg	< 0.01	< 0.0001 (mg kg^-1^ bw wk^-1^) ^†^	0.0016 (mg kg^-1^ bw wk^-1^) ^†^	0.00–0.06^g^
Pb	0.075(0.012)	< 0.0001 (mg kg^-1^ bw wk^-1^) ^†^	0.025 (mg kg^-1^ bw wk^−1^) ^†^	≤ 0.07^cfbeg^

*Calculated amount of element which is likely to be consumed per day in palm oil, based upon an average of 37.1 g day^−1^ vegetable oil consumed^62^, assuming palm oil is the primary vegetable oil in the diet. This is the average daily consumption of vegetable oil in China, one of the world’s largest palm oil importers.

^+^Values for males and females 19–70 years of age^61^. Refers to the amount that can be consumed daily by most individuals with no adverse health effects.

^†^Values for Al, Cd, Hg and Pb are in mg kg^−1^ bw week^−1^ due to these elements having a specified tolerable weekly intake rather than a tolerable upper limit^65–67^. The calculated maximum consumed quantity assumes an average body weight of 70 kg. Persons under 41 kg may be at risk of consuming above the maximum weekly intake of Al if consuming ≤ 37.1 g of palm oil per day.

^‡^Recommended dietary allowance as no tolerable upper limit could be identified. Refers to the daily intake that is sufficient for meeting nutrient requirements in 97–98% of the general population ^61^. Applies to non-pregnant or lactating females and males aged 9 – > 70 years.

^a^^58^.

^b^^59^.

^c^^22^.

^d^^68^.

^e^^60^.

^f^^21^.

^g^^69^.

^h^^70^.

^i^^71^.

^j^^72^.

The following elements were present in our analysed palm oil at concentrations comparable to or less than concentrations in palm oils from previous studies: Mg, P, K, Ca, Cr, Mn, Fe, Co, Ni, Cu, Zn, As, Cd, Hg and Pb (Table 6). Concentrations of Na and Al in our palm oil samples were higher than previously reported. The mean concentration of Na in our samples was 1198 mg kg^-1^. This is higher than reported levels in Nigerian palm oils of 30 mg kg^-1^^58^ and 115–533 mg kg^-1^^59^, however, would not result in consumption beyond the tolerable upper limit of 2300 mg Na day^-1^. The mean concentration of Al in our palm oil samples (159 mg kg^-1^) was also higher than those previously reported in Nigerian palm oil by Obi et al.^58^ (31 mg kg^-1^) and Asemave et al.^60^ (1.9 mg kg^-1^), however below concentrations which would surpass its tolerable weekly intake of 1 mg kg^-1^ bw wk^-1^, assuming an average body weight of 70 kg^61^ and an average daily palm oil intake of 37.1 g^62^. For people weighing < 41 kg, exceedance of the tolerable weekly intake may occur if they are consuming ≤ 37.1 g of palm oil per day, the average vegetable oil consumption in China—a major importer of palm oil. High Al and Na in plant derived products are usually attributed to soil or dust contamination of plant materials (e.g., during harvesting)^63^, however as these elements were found in the oil they may also come from processing. Other potential reasons for higher concentrations of these elements in the samples analysed include the use of saline washdown water in mills. The high Al concentrations in palm oil are supported by the mean Al concentrations in mesocarp samples (28–35 mg kg^-1^). Unlike most other TEs, Al may form hydrophobic organic complexes in plants which are phloem mobile, thereby partitioning into palm oil in the mesocarp^64^.

## Conclusions

The *E. guineensis* plant materials from North Sumatra, Indonesia, were deficient in essential elements including N, P, K and Mo, likely reducing palm oil yields from these sites. This is consistent with our hypotheses that *E. guineensis* leaves will show nutrient deficiencies, however, Mg was not deficient in leaf tissue as we had hypothesised. Leaf tissue from all sites was deficient in K, indicating that K may have been a yield-limiting factor potentially leading to the abandonment of production at two of the sites sampled. There is clear evidence that element deficiencies contribute to site abandonment. Leaf tissues showed no sign of elevated Cu, Zn, As and Pb, which falsified our hypothesis that leaves may contain elevated levels of TEs. Copper, however, had BACs > 1, indicating either elevated plant uptake or surface contamination from Cu-containing agrichemicals. This may result in *E. guineensis*-based products that exceed food and fodder safety standards if plants are grown on soils with excessive Cu concentrations. Palm oil generally contained low concentrations of TEs which were not likely to pose any toxicity risks. This was consistent with our hypothesis and with plant physiology considering trace elements often occur in plants as metal complexes which partition away from the oil phase. Aluminium and Na were present in palm oil at higher concentrations than had been previously reported in other studies, however, were not in breach of food safety standards. Future work could assess the bioaccumulation coefficients of TEs in *E. guineensis* under a range of conditions to determine whether plant tissues or oils may exceed toxicity thresholds or food safety standards with large fertiliser applications. This could be achieved in a dose–response pot trial. This would inform reasonable limits for fertiliser applications and contaminants to limit the transfer of TEs to plants and products in palm oil production systems.

### Supplementary Information

Supplementary Information.

## Data Availability

The data that support the findings of this study are openly available at www.kiwiscience.com/Data/Thompson-Morrison-PlantPaperData.xlsx.

## References

[1] Shahbandeh, M. (2022). *Consumption of vegetable oils worldwide from 2013/14 to 2021/2022, by oil type*. Statista. Retrieved 9th May, 2022, from https://www.statista.com/statistics/263937/vegetable-oils-global-consumption/

[2] OEC. (2020). *Palm oil*. Retrieved 17th May, 2022, from https://oec.world/en/profile/hs/palm-oil#:~:text=In%202020%2C%20the%20top%20exporters,and%20Spain%20(%241.25B).

[3] Vijay V, Pimm SL, Jenkins CN, Smith SJ (2016). The impacts of oil palm on recent deforestation and biodiversity loss. PLoS ONE.

[4] Foy, C. D. (1984). Physiological effects of hydrogen, aluminum, and manganese toxicities in acid soil. In F. Adams (Ed.), *Agronomy monograph*. 10.2134/agronmonogr12.2ed.c2

[5] Hartemink, A. E. (2002). Soil science in tropical and temperate regions—Some differences and similarities. In D. L. Sparks (Ed.), *Advances in Agronomy* (Vol. 77, pp. 269–292). Academic Press. https://www.sciencedirect.com/science/article/pii/S006521130277016810.1016/S0065-2113(02)77016-8

[6] Ng SK, Pasricha NS, Bansal SK (2002). Nutrition and nutrient management of oil palm—New thrust for the future perspective. Potassium for sustainable crop production: International symposium on the role of potassium.

[7] Woittiez LS, Slingerland M, Giller KE (2015). Yield gaps in Indonesian smallholder plantations: Causes and solutions International Palm Oil Congress and Exhibition.

[8] Chaney RL, Bar-Yosef B, Barrow NJ, Goldshmid J (1989). Toxic element accumulation in soils and crops: protecting soil fertility and agricultural food chains. Inorganic contaminants in the vadose zone.

[9] Aderungboye FO (1977). Diseases of the oil palm. PANS.

[10] MPOB. *Chemical fertiliser Ganocare™ as preventive treatment in controlling ganoderma disease of oil palm* (Malaysian Palm Oil Board TT No. 564) (2015). http://palmoilis.mpob.gov.my/publications/TOT/TT564-Idris.pdf

[11] Rys, G. J. *A national cadmium management strategy for New Zealand agriculture*. Cadmium Working Group (2011).

[12] Marschner, H. *Mineral nutrition of higher plants*. Gulf Professional Publishing (1995).

[13] Kabata-Pendias A, Mukherjee AB (2007). Trace elements from soil to human.

[14] Corley, R. H. V., & Tinker, P. B. H. *The oil palm*^(^5th ed.). Wiley (2015). https://books.google.co.nz/books?id=71A1CAAAQBAJ

[15] Kabata-Pendias, A., & Pendias, H. (2001). *Trace elements in soil and plants* (3rd ed.). CRC Press.

[16] Hooda P (2010). Trace elements in soils.

[17] Von Uexküll, H. R., & Fairhurst, T. H. *Fertilising for high yield and quality: The oil palm*. International Potash Institute (1991). https://www.ipipotash.org/uploads/udocs/ipi_bulletin_12_fertilizing_for_high_yield_and_quality_the_oil_palm.pdf

[18] Salisbury, F. B., & Ross, C. W. *Plant physiology* (4th ed.). Wadsworth, Inc (1992).

[19] Thompson-Morrison H, Gaw S, Robinson B (2022). An assessment of trace element accumulation in palm oil production. Sustainability.

[20] Gupta, M. *Practical guide to vegetable oil processing*. Elsevier Science (2017). https://books.google.co.nz/books?id=Ypp_CwAAQBAJ

[21] Rossi, M., Gianazza, M., Alamprese, C., & Stanga, F. The role of bleaching clays and synthetic silica in palm oil physical refining. *Food Chemistry, 82*(2), 291–296 (2003). 10.1016/S0308-8146(02)00551-4

[22] Szydłowska-Czerniak, A., Trokowski, K., Karlovits, G., & Szłyk, E. Spectroscopic determination of metals in palm oils from different stages of the technological process. *J. Agric. Food Chem. 61*, 2276−2283 (2013). 10.1021/jf305094s 10.1021/jf305094s 23394464

[23] Thompson-Morrison H, Ariantiningsih F, Arief SM, Gaw S, Robinson B (2023). Nutrients and contaminants in soils of current and former oil palm production systems from Indonesia. Land.

[24] Encyclopedia Britannica. (2016). Acrisol. https://www.britannica.com/science/Acrisol

[25] US EPA. *Method 3051A (SW-846): Microwave assisted acid digestion of sediments, sludges, soils, and oils, Revision 1*. US EPA (2007).

[26] Milestone. (n.d.). *Digestion method for environmental samples*. Milestone.

[27] INCT. *Polish certified reference material for multielement trace analysis: Oriental basma tobacco leaves (INCT-OBTL-5)*. Institute of Nuclear Chemistry and Technology (2010).

[28] NIST. *Certificate of analysis: Standard reference material 1573a tomato leaves*. National Institute of Standards & Technology (1995).

[29] NIST. *Certificate of analysis: Standard reference material 1643f trace elements in water* (2015). National Institute of Standards & Technology.

[30] LECO. *Instrument: CNS928 determination of carbon, nitrogen and sulfur in plant tissue*. LECO corporation (2019). https://eu.leco.com/images/Analytical-Application-Library/CNS928_CNS_PLANT_TISSUE_203-821-592.pdf

[31] Koleli, N., Demir, A., Kantar, C., Atag, G. A., Kusvuran, K., & Binzet, R. Heavy metal accumulation in serpentine flora of Mersin-Findikpinari (Turkey)—role of ethylenediamine tetraacetic acid in facilitating extraction of nickel. In K. R. Hakeem, M. Sabir, M. Öztürk, & A. R. Mermut (Eds.), Soil remediation and plants (pp. 629–659). Academic Press (2015). https://www.sciencedirect.com/science/article/pii/B978012799937100022X10.1016/B978-0-12-799937-1.00022-X

[32] Mishra, T., & Pandey, V. C. Phytoremediation of red mud deposits through natural succession. In V. C. Pandey & K. Bauddh (Eds.), Phytomanagement of polluted sites (pp. 409–424). Elsevier (2019). https://www.sciencedirect.com/science/article/pii/B978012813912700016810.1016/B978-0-12-813912-7.00016-8

[33] Taylor, M., Kim, N., Smidt, G., McNally, S., Robinson, B., Kratz, S., & Schnug, E. Trace element contaminants and radioactivity from phosphate fertiliser. In E. Schnug & L. J. De Kok (Eds.), Phosphorus in agriculture: 100% zero (pp. 231–266) (2016). Springer. 10.1007/978-94-017-7612-7_12

[34] R Core Team. *R: A language and environment for statistical computing* (2021). https://www.R-project.org/.

[35] Hothorn T, Bretz F, Westfall P (2008). Simultaneous inference in general parametric models. Biometr. J..

[36] Rafflegeau S, Michel-Dounias I, Tailliez B, Ndigui B, Papy F (2010). Unexpected N and K nutrition diagnosis in oil palm smallholdings using references of high-yielding industrial plantations. Agron. Sustain. Dev..

[37] Esperschuetz J, Anderson C, Bulman S, Katamian O, Horswell J, Dickinson NM, Robinson BH (2017). Response of Leptospermum scoparium, Kunzea robusta and Pinus radiata to contrasting biowastes. Sci. Total Environ..

[38] McLaren, R. G., & Cameron, K. C. *Soil ccience: Sustainable production and environmental protection*. Oxford University Press (1996). https://books.google.co.nz/books?id=AYVFAQAAIAAJ

[39] Chew, P. S., & Teoh, K. C. Use of rachis analysis an indicator of K nutrient status in oil palm. International Oil Palm/Palm Oil Conference: Progress and Prospects, Kuala Lumpur (1987).

[40] Cui J, Lamade E, Tcherkez G (2020). Potassium deficiency reconfigures sugar export and induces catecholamine accumulation in oil palm leaves. Plant Sci..

[41] Rankine I, Fairhurst TH (1999). Management of phosphorus, potassium and magnesium in mature oil palm. Better Crops Int..

[42] Jacquemard, J. C., H. Edyana, S., Dadang, K., & Tailliez, B. Expression of boron deficiency symptoms and link with the genotype in oil palm (Elaeis guineensis Jacq.) (2006). IOPC, Bali, Indonesia.

[43] Aini Azura A, Fauziah CI, Samsuri AW (2012). Cadmium and zinc concentrations in soils and oil palm tissues as affected by long-term application of phosphate rock fertilizers. Soil Sediment Contam. Int. J..

[44] El-Jaoual T, Cox DA (1998). Manganese toxicity in plants. J. Plant Nutr..

[45] Mason, B. H., & Moore, C. B. (1982). *Principles of geochemistry*. Wiley. https://books.google.co.nz/books?id=K75gIAAACAAJ

[46] Saleeb N, Gooneratne R, Cavanagh J, Bunt C, Hossain AKMM, Gaw S, Robinson B (2019). The mobility of silver nanoparticles and silver ions in the soil-plant system. J. Environ. Qual..

[47] Cowgill UM (1988). The tellurium content of vegetation. Biol. Trace Elem. Res..

[48] Akpanabiatu MI, Ekpa OD, Mauro A, Rizzo R (2001). Nutrient composition of Nigerian palm kernel from the dura and tenera varieties of the oil palm (Elaeis guineensis). Food Chem..

[49] Kok S, Ong-Abdullah M, Ee GC, Namasivayam P (2011). Comparison of nutrient composition in kernel of tenera and clonal materials of oil palm (*Elaeis guineensis* Jacq.). Food Chem..

[50] Mathews J, Lee AK, Clarence PJ, Chung MY, Rao S (2004). Oil content in oil palm fruit mesocarp and bunch, and some of its related physiological and agronomical factors. The Planter.

[51] Singh, P. K., Pratap, S. G., & Tandon, P. K. The mechanisms of trace element uptake and transport up to grains of crop plants. In Kumkum Mishra, Pramod Kumar Tandon, & S. Srivastava (Eds.), *Sustainable solutions for elemental deficiency and excess in crop plants* (pp. 119–133). Springer Singapore (2020).. 10.1007/978-981-15-8636-1_6

[52] Aladesanmi OT, Oroboade JG, Osisiogu CP, Osewole AO (2019). Bioaccumulation factor of selected heavy metals in Zea mays. J. Health Pollut..

[53] Reiser R, Simmler M, Portmann D, Clucas L, Schulin R, Robinson B (2014). Cadmium concentrations in New Zealand pastures: Relationships to soil and climate variables. J. Environ. Qual..

[54] Chalil, D. Market power and subsidies in the Indonesian palm oil industry AARES 52nd Annual conference, Canberra, Australia (2008). 10.22004/ag.econ.602210.22004/ag.econ.6022

[55] Ouerdane L, Mari S, Czernic P, Lebrun M, Łobiński R (2006). Speciation of non-covalent nickel species in plant tissue extracts by electrospray Q-TOFMS/MS after their isolation by 2D size exclusion-hydrophilic interaction LC (SEC-HILIC) monitored by ICP-MS. J. Anal. At. Spectrom..

[56] Almeida M, Carpenter K (2015). Determining trace elements in edible oils using inductively coupled plasma–optical emission spectrometry. Spectrosc. Suppl. Special Issues.

[57] Perlein A, Zdanevitch I, Gaucher R, Robinson B, Papin A, Sahraoui AL-H, Bert V (2021). Phytomanagement of a metal(loid)-contaminated agricultural site using aromatic and medicinal plants to produce essential oils: analysis of the metal(loid) fate in the value chain. Environ. Sci. Pollut. Res..

[58] Obi AL, Jonah SA, Umar I (2001). Determination of trace elements in some Nigerian vegetable based oilsby neutron activation analysis. J. Radioanal. Nucl. Chem..

[59] Nnorom IC, Alagbaoso JE, Amaechi UH, Kanu C, Ewuzie U (2014). Determination of beneficial and toxic metals in fresh palm oil (E*laeis guineensis* Jacq.) from south-eastern Nigeria: Estimation of dietary intake benefits and risks. J. Sci. Res. Rep..

[60] Asemave K, Ubwa ST, Anhwange BA, Gbaamende AG (2012). Comparative evaluation of some metals in palm oil, groundnut oil and soybean oil from Nigeria. Int. J. Mod. Chem..

[61] Otten, J. J., Hellwig, J. P., & Linda D. Meyers. (2006). *Dietary reference intakes: The essential guide to nutrient requirements*. National Academies Press. https://www.nal.usda.gov/sites/default/files/fnic_uploads/DRIEssentialGuideNutReq.pdf

[62] Zhao X, Xiang X, Huang J, Ma Y, Sun J, Zhu D (2021). Studying the evaluation model of the nutritional quality of edible vegetable oil based on dietary nutrient reference intake. ACS Omega.

[63] Hinton, T.G., Kopp, P., Ibrahim, S., Bubryak, I., Syomov, A., Tobler, L., Bell, C., 1995. A comparison of techniques used to estimate the amount of resuspended soil on plant surfaces. *Health Phys.* **68**, 523e531. 10.1097/00004032-199504000-00009 7883564

[64] Benning, U., Tamot, B., Guelette, B., & Hoffmann-Benning, S. (2012, 2012-March-28). New aspects of phloem-mediated long-distance lipid signaling in plants [Hypothesis and Theory]. *Front. Plant Sci*. 10.3389/fpls.2012.00053 10.3389/fpls.2012.00053 PMC3355628 22639651

[65] Aguilar F, Autrup H, Barlow S, Castle L, Crebelli R, Dekant W, Engel K-H, Gontard N, Gott D, Grilli S, Gürtler R, Larsen J-C, Leclercq C, Leblanc J-C, Malcata F-X, Mennes W, Milana M-R, Pratt I, Rietjens I, Tobback P, Toldrá F (2008). Safety of aluminium from dietary intake - scientific opinion of the panel on food additives, flavourings, processing aids and food contact materials (afc). EFSA J..

[66] FAO/WHO. (2000). *Safety evaluation of certain food additives and contaminants* (WHO Food Additives Series, Issue. WHO/FAO. https://inchem.org/documents/jecfa/jecmono/v44jec12.htm

[67] FAO/WHO. (2004). *Evaluation of certain food additives and contaminants* (WHO Techncial Report Series—922, Issue. WHO. https://apps.who.int/iris/bitstream/handle/10665/42849/WHO_TRS_922.pdf?sequence=1&isAllowed=y 15354533

[68] Njoku PC, Egbukole MO, Enenebeaku CK (2010). Physio-chemical characteristics and dietary metal levels of oil from elaeis guineensis species. Pak. J. Nutr..

[69] Adepoju-Bello AA, Osagiede SA, Oguntibeju OO (2012). Evaluation of the concentration of some toxic metals in dietary red palm oil. J. Bioanal. Biomed..

[70] Aigberua AO, Ovuru KF, Izah SC (2017). Evaluation of selected heavy metals in palm oil sold in some markets in Yenagoa metropolis, Bayelsa State, Nigeria. E C Nutr..

[71] Chen SS, Lee BY, Cheng CC, Chou SS (2001). Determination of arsenic in edible fats and oils by focused microwave digestion and atomic fluorescence spectrometer. J. Food Drug Anal..

[72] Chen SS, Cheng CC, Chou SS (2003). Determination of arsenic in edible fats and oils by direct grahite furnace atomic absorption spectrometry. J. Food Drug Anal..

