# Supplementary material for: Chemical elements in Elaeis guineensis materials and derived oil
Source: Sci Rep. 2024 Jan 22;14:1836. doi: 10.1038/s41598-023-50492-8 (PMC10800330; doi:10.1038/s41598-023-50492-8)
Supplement: Supplementary file 1 — Supplementary Information. [file 41598_2023_50492_MOESM1_ESM.pdf]

Chemical elements in *Elaeis guineensis* materials and derived oil

Hadee Thompson-Morrison [1*+], Fransisca Ariantiningsih [2], Sugesti Muhammad Arief [2], Sally Gaw [1], Brett Robinson [1]

[1] School of Physical and Chemical Sciences, University of Canterbury, Christchurch, New Zealand
[1] Orangutan Information Centre, Medan, Sumatra, Indonesia
[*] Corresponding author – address for correspondence: Thompson-MorrisonH@landcareresearch.co.nz
+ Current address: Manaaki Whenua – Landcare Research, Lincoln, New Zealand

Supplementary Materials

Table S1. SRM recoveries of elements

Element	SRM recovery of certified value (%)
**B**	105
**Na**	119
**Mg**	88
**Al**	79
**Si**	No SRM — concentrations were comparable to land plants average of 200 mg kg^{-1} (Mason & Moore, 1982)
**P**	95
**S**	134
**K**	98
**Ti**	No SRM — concentrations were comparable to land plants average of 1.0 mg kg^{-1} (Mason & Moore, 1982)
**Cr**	97
**Mn**	109
**Fe**	103
**Co**	99
**Co**	116
**Ni**	99
**Cu**	100
**Zn**	97
**As**	102
**Sr**	110
**Zr**	No SRM
**Mo**	98
**Ag**	134
**Cd**	94
**Te**	76
**Cs**	107
**Ce**	84
**Au**	153
**Hg**	97
**Pb**	No SRM — concentrations were comparable to land plants average of 2.7 mg kg^{-1} (Mason & Moore, 1982)

References

Mason, B. H., & Moore, C. B. (1982). *Principles of geochemistry*. Wiley. https://books.google.co.nz/books?id=K75gIAAACAAJ
